# The role of ambulatory 24‐hour esophageal manometry in clinical practice

**DOI:** 10.1111/nmo.13861

**Published:** 2020-05-11

**Authors:** Afrin N. Kamal, John O. Clarke, Jac M. Oors, Albert J. Bredenoord

**Affiliations:** ^1^ Division of Gastroenterology and Hepatology Stanford University School of Medicine Stanford CA USA; ^2^ Department of Gastroenterology and Hepatology Amsterdam University Medical Centre Amsterdam The Netherlands

**Keywords:** ambulatory esophageal manometry, non‐cardiac chest pain, reflux‐cough association, rumination syndrome

## Abstract

High‐resolution manometry revolutionized the assessment of esophageal motility disorders and upgraded the classification through the Chicago Classification. A known disadvantage of standard HRM, however, is the inability to record esophageal motility function for an extended time interval; therefore, it represents only a more snapshot view of esophageal motor function. In contrast, ambulatory esophageal manometry measures esophageal motility over a prolonged period and detects motor activity during the entire circadian cycle. Furthermore, ambulatory manometry has the ability to measure temporal correlations between symptoms and motor events. This article aimed to review the clinical implications of ambulatory esophageal manometry for various symptoms, covering literature on the manometry catheter, interpretation of findings, and relevance in clinical practice specific to the evaluation of non‐cardiac chest pain, chronic cough, and rumination syndrome.

## Graphical abstract

Ambulatory esophageal manometry measures esophageal motility over a prolonged period and detects motor activity during the entire circadian cycle. This article aims to review the clinical implications of ambulatory esophageal manometry for various symptoms, covering literature on the manometry catheter, interpretation of findings and relevance in clinical practice specific to the evaluation of non‐cardiac chest pain, chronic cough and rumination syndrome.

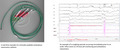

**Key Points**

- Ambulatory esophageal manometry measures esophageal motility over a prolonged period and has the ability to measure temporal correlations between symtoms and motor events.
- This article aims to review the clinical implications of ambulatory esophageal manometry in non‐cardiac chest pain, chronic cough, and rumination syndrome.
- The application of ambulatory esophageal manometry has mainly been a tool for experts’ centers and is not well known, but adds advantage over stationary manometry by evaluating esophageal function over an extended period of time and can add critical information to ambulatory pH‐impedance testing. Additional research will be important to further understand the value of ambulatory manometry in clinical practice.

## 1 INTRODUCTION

Esophageal manometry was first introduced in 1883 by Kronecker et al^1^ Today, high‐resolution manometry (HRM) is the primary method used to evaluate esophageal motor function, incorporating up to 36 pressure sensors, spaced 1 cm apart along a catheter. HRM with pressure topography has improved our ability to study esophageal motility and visualize both peristaltic and sphincter functions.^2^, ^3^ The advent of HRM has led to a change in the classification of esophageal motor disorders; the Chicago Classification (CC) was introduced in 2009 to define and characterize major, hypercontractile, and minor motility disorders.^4^

A disadvantage of standard HRM is the inability to record esophageal motility for an extended time interval because of a pressure drift that occurs with time and in ambulatory conditions. The standardized protocol (with both conventional manometry and HRM) is in supine position during which 10 wet swallows of boluses of 5 mL of water are completed with 30‐second intervals, following a 30‐second measurement of resting pressure. Occasionally, adjunctive tests will follow the standard protocol such as multiple rapid swallows.^4^ In total, the entire manometry protocol will typically last no longer than 30 minutes. As the catheter is connected to a large hardware system, ambulatory conditions are not possible. These time‐limited techniques to evaluate esophageal motility are not ideal when aiming to measure temporal correlations between symptoms and motor events. Furthermore, esophageal motor disorders associated with non‐cardiac chest pain (ie, distal esophageal spasm) and occasionally with dysphagia may occur only intermittently, and therefore may not be captured during a 30‐minute stationary assessment. In consequence, ambulatory 24‐hour esophageal manometry recording was introduced, mostly used in combination with ambulatory reflux monitoring. This article aimed to review the clinical implications of ambulatory esophageal manometry for various symptoms, covering literature on the manometry catheter, interpretation of findings, and relevance in clinical practice.

## 2 AMBULATORY 24‐HOUR ESOPHAGEAL MANOMETRY

**Figure 1:**
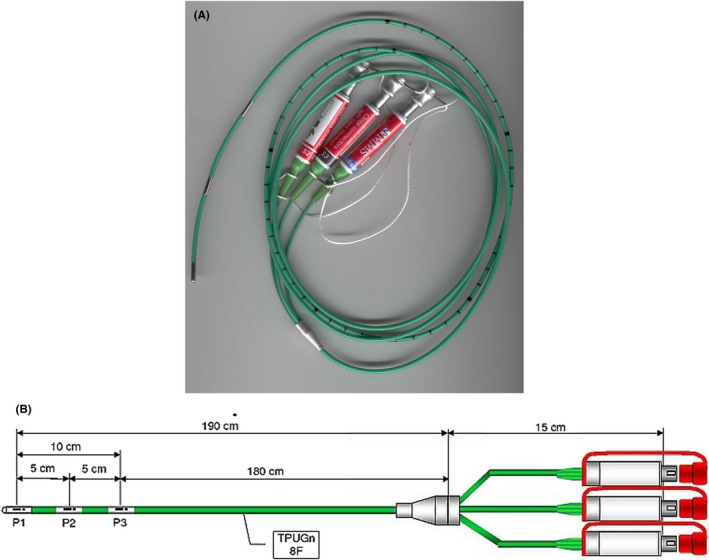
A and B, An illustrative and real‐time example of a clinically available ambulatory manometry catheter, demonstrating the catheter length and number of pressure sensors

In contrast to stationary time‐limited manometry, ambulatory esophageal manometry records measurements over a prolonged period—usually 24 hours. The catheter typically consists of a 5‐7 French (Fr) polyurethane probe with miniature pressure transducers able to measure pressure variations ranging between −50 and 350 mm Hg within the esophageal lumen (Figure 1A). Typically, the sensors are of the strain gauge force transducer type, consisting of resistors whose electrical resistance changes when a force is applied to it. The principle of measurement with these sensors is fundamentally different from that used in fiber optical pressure measurement systems, such as that used in most widely used HRM system (Manoscan, Medtronic). The latter is not suited for prolonged manometry because of drift. Ambulatory esophageal manometry can also be carried out with a perfused catheter, but this requires a cumbersome and not commercially available portable water‐perfusion system.^5^, ^6^

Before each use, the manometric system that consists of catheter and digital signal conditioning and recording device needs to be calibrated. This is usually done by submerging the catheter in a water‐filled calibrating tube, applying pressures of 0 and +50 mm Hg. All medications that may interfere with gastrointestinal motility (ie, prokinetics) are discontinued at least 48 hours prior and patients arrive following an overnight fast. With trans‐nasal introduction, the manometric sensors are positioned along the esophageal body. The exact placement of the transducers, however, may vary depending on indication. More commonly pressure sensors will be placed at 5, 10, and 15 cm proximal to the LES, but some protocols (ie, for rumination) will extend the catheter distally into the gastric lumen. It must be noted that the LES pressure cannot be recorded reliably in an ambulatory setting, unless the catheter is fitted with an array of closely spaced sensors that straddle the area. An important additional recognition will be the differences in the ambulatory manometry catheter among various authors. Currently, at our institution, a clinically available solid‐state 8‐Fr, 180‐cm length manometry catheter is used, composed of three pressure sensors spaced 5 cm apart (Laborie; Figure 1B).

Ambulatory pressure measurements are usually combined with ambulatory reflux monitoring with pH or pH‐impedance monitoring using a second separate catheter. Applying a menu‐driven graphic software tool, pressure and pH signals are displayed, together with information on body position (ie, supine), eating periods, and timing of symptoms. Following catheter placement, patients return home for the next 24 hours and report sleep and mealtimes, in addition to symptom events using the portable recording device. At completion of the measurement, patients return back, the catheter is removed, and the manometric data are transferred into a software program for analysis.^7^, ^8^, ^9^

## 3 DIFFERENCES BETWEEN STATIONARY AND AMBULATORY ESOPHAGEAL MANOMETRY

On average, during a 24‐hour continuous monitoring period, approximately 1000 to 1400 contractions are recorded by each transducer. An important difference with stationary esophageal manometry is that the frequency of contractions, duration, amplitude, and prevalence of peristaltic waves vary considerably during a normal circadian cycle. During nocturnal sleep, esophageal motor activity is significantly decreased, and the majority of contractions that occur are simultaneous and of high amplitude. In the awake condition, on the other hand, esophageal motor activity is frequent, between meals, with a predominance of peristaltic contractions.^9^ In the controlled situation of stationary manometry, patients are instructed to not move and are usually in the recumbent position. The only challenge used to test esophageal motor consists of 10 wet swallows. Whereas with ambulatory manometry, patients are encouraged to resume daily behaviors including being physically active, erect, or lying in varying positions. In addition, there are no standardized boluses in ambulatory manometry—most swallows are dry swallows. Therefore, conventional and HRM criteria of esophageal motor disorders are not applicable in ambulatory esophageal motility monitoring. For example, in the Spechler and Castell classification for esophageal motility disorders, diffuse esophageal spasm (DES) is defined as >20% simultaneous contractions, whereas in ambulatory manometry, the upper limit of normal is 55% simultaneous contractions upright, and 80% simultaneous contractions at night.^10^, ^11^

Another difference between stationary and ambulatory manometry is that the catheter for ambulatory manometry has a limited number of sensors that does not allow reliable measurement of LES resting and relaxation pressure, while this is the most important part of stationary manometry. As the larynx elevates and the esophagus shortens with swallowing, the LES moves in the cephalad direction. As a consequence, a single pressure sensor will drop out of the high‐pressure zone, giving the false impression of sphincter relaxation. Recognizing this challenge, a perfused sleeve sensor has been developed consisting of a 6‐cm‐long silicone rubber membrane that covers a segment of the manometry catheter. The sleeve sensor picks up the highest pressure exerted along the length of the membrane.^12^, ^13^ Another possibility to overcome this problem is to use high‐resolution manometry with sensors or side holes at 1‐cm intervals, at least in the area of the EGJ.^14^ These techniques have been exclusively used in research studies.

The differences between ambulatory and stationary esophageal manometry also lead to differences in normal values. Normative values for ambulatory manometric parameters at three levels of the esophagus among 25 healthy volunteers (mean age 30 years, range 22‐48 years) were reported by Bremner et al In their study, a 7‐Fr catheter with three transducers, each separated by 5 cm, and concomitant 24‐hour pH monitoring was used. Following 24 hours, data were downloaded and manually analyzed for contraction amplitude, duration, morphology, and speed of propagation. The authors reported that the frequency of contractions was increased when awake, highest during meals, and lowest during sleep. Contraction amplitude was found to increase during meals as well. Furthermore, peristaltic waves were found to vary during different physiologic states, such as awake, eating, upright, and sleeping.^15^ Specific to a Hispanic population, Awad et al aimed to establish normal values for motility patterns using stationary and 24‐hour esophageal manometry among 12 healthy volunteers. In their ambulatory studies, the authors used a catheter with 4 solid‐state transducers, the most distal of which was a circumferentially sensitive transducer that was positioned in the LES. The authors demonstrated that mean LES pressures were lower by stationary measurement when compared to 24‐hour ambulatory manometry (20.8 ± 11.2 mm Hg, CI 14.4‐27.2 and 51.3 ± 10.5 mm Hg, CI 45.3‐57.3, respectively), whereas esophageal contraction mean amplitude was similar between both procedures. Therefore, the authors suggested that physiologic motility patterns may differ when employing 24‐hour manometry, particularly when evaluating LES pressures and duration of contractions. Although significant differences in LES values were seen, one should recognize that the technique for measuring LES during ambulatory manometry was not reliable as it used just one sensor and is completely different from stationary measurement. In the former, the authors measured LES pressure by a rapid pull‐through technique, withdrawing at a speed of 1 cm/s while breathing was withheld.^16^ In contrast, in stationary manometry LES was measured without any additional movements.

## 4 NON‐CARDIAC CHEST PAIN

Non‐cardiac chest pain (NCCP) is the descriptive term used for recurrent angina‐like pain in patients in whom coronary heart disease was excluded with standard diagnostic evaluation. NCCP is a common presentation with an estimated prevalence of up to 25% in the general population.^17^ NCCP is thought to relate to either GERD, esophageal visceral hypersensitivity, or esophageal dysmotility.^17^, ^18^ To provide guidance in NCCP evaluation, commonly patients will undergo workup with stationary esophageal HRM and 24‐hour pH‐impedance monitoring, in an effort to identify esophageal spasm, achalasia, or reflux disease as a potential cause of the symptoms.

The most important contributor to NCCP is GERD, with an estimated prevalence ranging between 30% and 60%.^19^ Karlaftis et al found that NCCP was GERD‐related in 58% of a group undergoing combined impedance/pH monitoring and gastroscopy, with chest pain symptoms more prevalent postprandially (*P* < .05).^20^ Prakash et al assessed the advantage of a wireless ambulatory pH‐monitoring system to diagnose GERD in NCCP patients, comparing the usual 24 hours of recording vs the final outcome of 48 hours. The authors observed an increase in the proportion of abnormal acid exposure time and the likelihood of a positive reflux symptom relationship by extending the pH evaluation beyond the standard 24 hours.^21^

Less commonly, NCCP patients demonstrate esophageal dysmotility as the presumed etiology of symptoms, estimated around 30%. In a 1987 study by Katz et al, among 910 patients with NCCP, 70% had normal esophageal motility, whereas 14.4% demonstrated nutcracker esophagus, followed by non‐specific esophageal disorders in 10.8%. Diffuse esophageal spasm, achalasia, and hypertensive LES were found to be less common as cause of NCCP.^22^ It remains of course the question whether the finding of nutcracker esophagus or hypertensive LES is a sufficient explanation for the presence of the chest pain. Achem et al found in a review of 402 patients with NCCP that 10% of patients were identified with nutcracker esophagus and that 35% of these patients had concomitant pathologic reflux.^23^ Dekel et al investigated the Clinical Outcomes Research Initiative database, a registry encompassing more than 60 academic centers, and found that 70% of NCCP subjects had normal esophageal motility, whereas only 10% demonstrated non‐specific esophageal motor disorders on stationary manometry including nutcracker esophagus.^24^

The low yield of stationary manometry in patients with chest pain has prompted the question whether ambulatory manometry could be of additional value. The argument supporting the assessment of ambulatory manometry is that stationary, short‐term motility assessment merely provides a glimpse into a patient's esophageal behavior, while typical angina‐like pain is often infrequently present, with complete absence of symptoms during most the of time. Since the late 1980s, several studies using 24‐hour manometry have evaluated the diagnostic value of this technique in the management of NCCP (Table 1). In 1989, Soffer et al performed esophageal 24‐hour ambulatory pH and motility recording among patients with NCCP, following baseline short‐term manometry evaluation. Out of 20 patients, only one patient was thought to experience symptoms due to esophageal dysmotility during the 24‐hour measurement, and therefore, the authors viewed long‐term manometric evaluation as having low diagnostic yield.^25^ In 1993, WG Paterson et al summarized their experience applying standard Holter electrocardiographic (ECG) monitoring with ambulatory esophageal manometry and pH‐metry among patients with atypical chest pain. Following standard manometry, the authors recorded ambulatory esophageal pressures using a solid‐state catheter with sensor ports at 5, 10, and 15 cm proximal to the LES. The authors described that for a small percentage of chest pain episodes, a temporal correlation was seen with reflux or esophageal motor disorders.^26^

**Table 1:** Study characteristics and findings of ambulatory manometry in non‐cardiac chest pain

	Study year	Total number of patients studied	Ambulatory manometry catheter description	Frequency of esophageal dysmotility detected	Type of esophageal dysmotility detected	Percentile (%) chest pain experienced during 24‐h monitoring
Barret et al	2016	59	8‐Fr solid‐state catheter, 5, 10, and 15 cm proximal to LES	6.8%	Esophageal spasm	69.5%
Breumelhof et al	1990	44	5‐Fr polyurethane catheter, 5 and 15 cm proximal to LES	23.4%	Esophageal dysmotility	56.8%
Cameron et al	2006	37		2.7%	Esophageal spasm	89%
J Ooi et al	2016	17	Ultra‐thin high‐resolution solid‐state catheter	25%		71%
Lam et al	1992	41	Five and 15 cm proximal to LES	43%	Esophageal dysmotility	73%
Paterson et al	1993	25	3 sensor ports at 5, 10, and 15 cm proximal to LES	15%	Esophageal dysmotility	68%
Peters et al	1988	24	Probe with two pressure transducers, 5 cm apart, on a 4.5‐mm‐diameter catheter	12%	Esophageal dysmotility	91.7%
Soffer et al	1989	20	2‐mm catheter, LES and 5 cm proximal	8.6%	Esophageal dysmotility	75%

Abbreviations: DES, diffuse esophageal spasm; Fr, French; LES, lower esophageal sphincter.

Years following, Lam et al performed 24‐hour pH and esophageal pressure recordings among 41 patients admitted for acute chest pain. Abnormal esophageal motility with pain was defined as a contraction amplitude or duration exceeding the patient's own upper limits of normal or an increased incidence of abnormally propagated contractions (ie, non‐transmitted, simultaneous). About 73% of patients experienced chest pain during the extended recording, 43% of these painful episodes were associated with abnormal esophageal motility. Applying a positive criterion of SI ≥ 75%, where SI reflected percentage of symptoms occurring immediately following dysmotility or reflux events, the authors found that pain was related to reflux in 13 (43%) patients and esophageal dysmotility in 10 (33%). Therefore, the authors concluded that in the majority NCCP patients, reflux or esophageal motor abnormalities were not the cause of the pain.^27^

Similarly, Peters et al assessed 24 patients with chest pain, applying 24‐hour ambulatory esophageal motility and pH monitoring. Abnormal motility was defined by exceeding the patient's normal mean amplitude and duration, maximum amplitude and duration, or percentage of abnormal peristalsis. Among the 24% of patients who experienced chest pain during the assessment, 11 (12%) episodes were temporally associated with abnormal motility and 18 (20%) with pH drop <4. Interestingly, the majority (64%) of symptoms lacked any presence of dysmotility or pH drop association.^28^ With the addition of edrophonium provocation, Breumelhof et al measured esophageal pressure and pH signals among 44 NCCP patients, applying 97.5th percentile of amplitude and duration of all esophageal contractions plus a chi‐square distribution of contraction types to assess an esophageal dysmotility relationship. Among the 56.8% of patients who had at least one pain episode during extended recording, 23.4% of the chest pain episodes were related to esophageal dysmotility, whereas 43.2% were entirely unrelated to esophageal function.^29^

Recognizing that 27%‐43% of patients undergoing ambulatory manometry do not experience chest pain during the 24‐hour testing and that the overall percentage of identified dysmotility has been low, routine usage of ambulatory esophageal manometry in clinical practice has been questioned.^30^ However, despite these disadvantages, ambulatory esophageal manometry is the only method to assess temporal relationships between symptoms of chest pain and dysmotility events; therefore, it continues to be utilized in certain centers. Most importantly, a negative test during a measurement period in which a symptom of chest pain was reported is very convincing to both physician and patient that the symptoms are not due to dysmotility but are more likely to be functional in nature. Such a finding will prevent the chronic but pointless use of calcium antagonists and nitrates for symptoms that are not spastic in nature and may direct the treatment to tricyclic antidepressants and hypnotherapy.

## 5 CHRONIC COUGH

**Figure 2:**
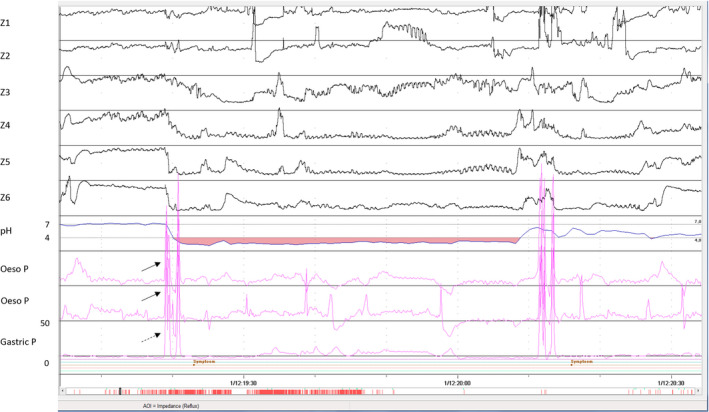
An example of a coughing episode occurring immediately prior to an acidic reflux event on 24‐h pH monitoring and ambulatory manometry. Cough is depicted by the acute simultaneous rise in gastric (broken arrow) and esophageal (solid arrow) pressure sensors

Accounting for an estimated 30 million clinical visits per year, chronic cough is a difficult diagnosis to elucidate. Etiologies for chronic cough range from upper airway disease, non‐asthmatic eosinophilic bronchitis, drug side effects, postnasal drip, and GERD.^31^ The clinical question is whether in a specific patient the symptoms of chronic cough are induced by reflux. The presence of excessive reflux on pH monitoring or endoscopic signs of reflux esophagitis does not prove that reflux is a causative factor. To investigate causality, analysis of the temporal relationship between cough and reflux is required. Ambulatory manometry makes this possible as cough can be clearly identified on the tracing, allowing an assessment of the reflux‐cough sequence during prolonged reflux monitoring. Of course, this can also be done with acoustic cough markers, but correct and precise interpretation of the timing of cough episodes and reflux events is essential. For example, if a cough is immediately followed by reflux, cough is likely leading to the reflux (Figure 2). On the other hand, if the cough occurs a few seconds following the onset of the reflux event, the suggestion is that reflux induces the cough. That being said, the question lingers whether there truly is a need to record the cough events objectively if a patient already reports cough symptoms in a diary? Many argue yes, as symptom reports are a reflection of patient compliance and can be subject to bias and there can easily be a delay of 30 seconds to a minute between symptom occurrence and noting this in the diary.^32^ For example, studies have demonstrated that pressure monitoring detects 70%‐90% more coughs than standard symptom report.^33^, ^34^ In order to improve cough detection, therefore, ambulatory manometry has been incorporated with reflux testing to detect acute pressure changes associated with cough, eliminating the possible bias of patient self‐reporting and therefore improving accuracy.^35^

Detection of cough on ambulatory manometry usually relies on pressure changes in both the esophageal and gastric lumen. The act of coughing is the coordinated effort of thoracic, abdominal, and pelvic muscle contraction. As a result of contracting external abdominal obliques, intercostals, and associated respiratory muscles, the diaphragm displaces superiorly with a concomitant rise in intra‐abdominal pressure.^36^ This gives rise to a rapid simultaneous increase in intrathoracic and abdominal pressure. In a multi‐center study of 49 patients, Herregods et al applied 24‐hour pH‐impedance/pressure monitoring to characterize reflux episodes followed by cough. Reflux episodes that were followed by a cough burst were more likely to have a higher proximal extent (*P* = .0001), higher volume clearance time (*P* = .002), and a larger acid burden within a 15‐minute window (*P* = .019), when compared to isolated reflux episodes.^37^

The application of ambulatory manometry in cough assessment was used in a number of studies that assessed the role of reflux. Bogte et al retrospectively reviewed patients with unexplained cough who underwent ambulatory 24‐hour pH and pressure monitoring. They aimed to assess temporal relationships between cough and reflux events within a 2‐minute time window, distinguishing whether cough was induced by reflux, coined “reflux‐cough sequence” (ie, cough occurred within 2 minutes after reflux episode) vs cough precipitating reflux referred as “cough‐reflux sequence” (ie, reflux occurs within 2 minutes immediately after a cough). Among 37 subjects coughing during the study, pathologic esophageal acid exposure was seen in 40.5%, a positive reflux‐cough sequence in 20%, and a positive cough‐reflux sequence in 13.5%. It is important to note the lack of a gastric pressure transducer utilized in this protocol, instead of detecting a coughing episode by symptom reports and simultaneous rise in esophageal pressure. The authors concluded that the application of combined 24‐hour pH and pressure monitoring may be clinically useful in distinguishing reflux‐cough relationships.^38^

Furthermore, to assess whether ambulatory manometry could help identify patients whose cough symptoms may benefit most from antireflux treatment, Sifrim et al measured the relationship between chronic cough and acidic reflux among 22 patients. The authors applied 24‐hour pH‐impedance monitoring and manometry with four solid‐state pressure sensors (Unisensor AG), positioned in the stomach, LES, and at 5 and 10 cm proximal to the LES. Subjects went back home and were encouraged to maintain usual meals and daily activities, while keeping a diary of cough events. The authors defined a “cough burst” as ≥2 rapid simultaneous pressure peaks occurring within 3 seconds, and subjects with <5 cough bursts within 24 hours were subsequently excluded. Study findings revealed majority of cough events (69.4%) were unrelated to reflux, whereas 30.6% took place within 2 minutes of a reflux episode. Of these coughing episodes around reflux events, almost half (49.0%) occurred after a reflux event (reflux‐cough), whereas the other half occurred before a reflux event (51.0%). Among the 22 patients, 45% were found to have a positive SAP between reflux and cough. The authors subsequently concluded that ambulatory pressure/pH‐impedance monitoring may provide relevant data that can affect diagnosis and therapeutic decision making, as it can reliably identify those patients whose cough symptoms may benefit from more aggressive antireflux treatment.^39^ In an even larger study, Blondeau et al measured the relationship between reflux and cough among 100 patients applying simultaneous ambulatory manometric‐impedance‐pH system. The manometric catheter consisted of two solid‐state pressure sensors, with one pressure channel positioned 5 cm proximal to the lower esophageal sphincter. On manometric tracing, the authors defined a single as a simultaneous phasic, short duration, rapid pressure rise, whereas a cough burst described ≥2 rapid simultaneous pressure peaks within 3 seconds. Applying ambulatory manometry technology, the authors observed patients experiencing reflux resulting in cough, in addition to cough inducing reflux.^40^ Of course, *for the purpose of detection of cough bursts measurement of intragastric pressure is required, measurement of esophageal pressure is less important for this indication. Strictly speaking, this is therefore not ambulatory esophageal manometry but ambulatory intragastric manometry. Furthermore* , measuring cough and reflux sounds simple, but can become complex with increasing number of cough episodes. In contrast to chest pain and heartburn, patients with chronic cough typically can experience several hundreds of coughs during a 24‐hour period, making the task to interpret recorded data difficult and time‐consuming.^41^

## 6 RUMINATION SYNDROME

The term rumination derives from the Latin word “ruminare,” meaning chewing the cud. Whereas the act of rumination is a normal digestive act among the subgroup of mammals known as ruminants, including cattle, sheep, and goats, the act of recurrent regurgitation of undigested food into the mouth, rechewing, swallowing, or spitting among humans is considered abnormal and it characterizes what is coined rumination syndrome.^42^ Rumination is thought to be a behavioral response, typically occurring 1‐2 hours following meals. Although the cause of rumination is unclear, it is believed to be an unconscious learned disorder characterized by a rapid increase in intragastric pressure. As gastric pressure rises beyond the LES pressure, stomach contents flow into the esophageal lumen and consequently through the UES as it relaxes. Consequently, gastric content flows into the pharynx and into the mouth and is spitted out or swallowed again.^43^ Unfortunately, rumination is frequently misdiagnosed as GERD, with initial diagnosis relying heavily on a detailed history. Currently, the clinical diagnosis is based on the fourth version of the Rome Criteria, defined by (a) persistent or recurrent regurgitation of recently ingested food and subsequent spitting or mastication and swallowing and (b) regurgitation not preceded by retching for the past 3 months.^44^

The clinical application of objective testing to diagnose rumination syndrome has been applied to support the clinical diagnosis. Such testing includes electromyography (EMG) of the anterior abdominal wall and intercostal muscles, stationary high‐resolution impedance manometry, and 24‐hour pH impedance combined with ambulatory esophageal manometry.^44^ Manometry has introduced the advantage of distinguishing rumination objectively from belching/regurgitation disorders and GERD, and by combining manometry with pH impedance, a new avenue of distinguishing the 3 subtypes of the syndrome has been identified.

**Figure 3:**
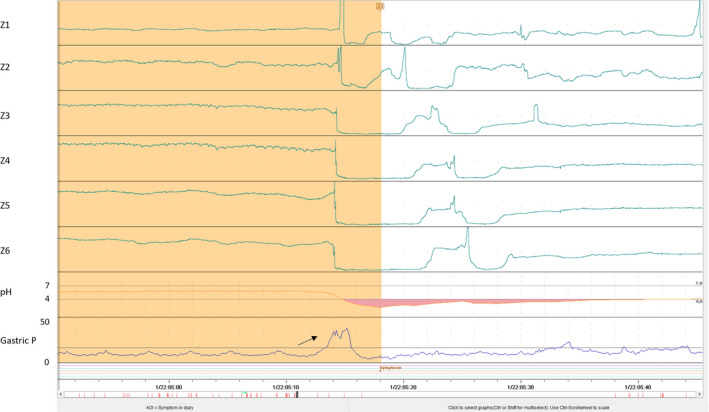
Rumination variants are measured by combined ambulatory manometry and pH impedance. Solid arrow signals the immediate rise in gastric pressure prior to a drop in impedance followed by a subsequent return of impedance back to baseline

The presentation of rumination on manometry has been identified as a sharp rise in intragastric pressure (gastric strain), referred to as the “R‐wave” (Figure 3). On the other hand, supragastric rumination is seen by ingested air by impedance and subsequent rise in intragastric pressure causing flow of esophageal contents into the mouth, whereas reflux‐rumination is seen as a gastric strain following a transient LES relaxation. In the end, a key component to rumination is manometric evidence of a rise in intragastric pressure immediately preceding the start of flow of gastric contents into the esophagus.^45^ Ambulatory manometry provides the advantage of assessing rumination in the home setting, as patients may not ruminate in the short‐term setting of stationary esophageal manometry. Furthermore, as the differentiation of rumination is often GERD, combined ambulatory pH impedance provides this additional assessment.

The manometric characteristics of the different rumination symptoms were evaluated by Kessing et al as the group reviewed 96 symptom episodes during ambulatory manometry/impedance monitoring and 37 symptom episodes on high‐resolution manometry/impedance (HRIM) among 5 patients with clinically defined rumination. The protocol consisted of consuming a standardized liquid meal followed by stationary HRIM measurements and subsequent 24‐hour ambulatory manometry/impedance monitoring. Rumination events were seen on HRIM in 32 out of 37 symptom episodes (SI 86%), with UES relaxation during all events. Ambulatory measurements confirmed rumination events in 85 out of 96 symptom episodes (89%), all occurring in the upright position and 90% within the postprandial period. In 60% of rumination events, the characteristic intragastric pressure rise occurred prior to the retrograde flow of esophageal fluid and simultaneously in 40% of events. The authors concluded that the combination of both stationary high‐resolution and ambulatory manometry allowed for a more detailed description and identification of rumination events.^46^ Supporting criteria for rumination are predominant postprandial reflux events, rapidly repetitive reflux events, reflux events reaching the most proximal impedance channel, and absence of these events during the night.^47^

Kessing et al measured ambulatory manometry and pH‐impedance measurements following completion of a standard HRM protocol. The solid‐state ambulatory manometry catheter consisted of four pressure sensors, positioned 5 cm distal to the LES and 5, 10, and 15 cm proximal to the LES border. Subsequent impedance, pH, and manometry measurements were recorded over 24 hours. In all rumination patients, multiple proximal reflux events were associated with a pressure peaks >30 mm Hg, and 57% of total rumination episodes occurred with a concomitant increase in gastric pressure >30 mm Hg. Therefore, the authors suggested diagnosis could be made with the presence of multiple reflux events extending into the proximal esophagus and an associated rise in intragastric pressure >30 mm Hg.^48^

Ambulatory manometry provides additional advantages in identifying rumination among children. Rumination syndrome is common in pediatrics. In an epidemiology study assessing prevalence and symptomatology among school children, Rajindrajith et al observed among 110 children, 62.7% reported symptoms once per week, whereas only 8.2% experienced daily symptoms.^49^ These sporadic rumination events introduce challenges when stationary manometry is limited to a 30‐minute assessment, at best. The application of 24‐hour pH‐impedance monitoring and manometry as a diagnostic tool in rumination syndrome among children was assessed by Singendonk et al The authors reviewed records of children suspected with rumination, evaluating degree of retrograde bolus flow extending into the proximal esophagus in addition to peak gastric and intra‐esophageal pressures at time of recorded symptoms. Among 18 children with suspected rumination, rumination events were seen in 88.9%, with 50% seen at <30 minutes postprandially. Among the 16 children with confirmed rumination, majority of subjects (93.8%) demonstrated ≥1 gastric pressure peak >30 mm Hg. The authors therefore felt encouraged combined 24‐hour pH‐impedance monitoring and manometry could be applied to diagnose rumination syndrome among children. Furthermore, they proposed a cutoff for gastric pressure increase >25 mm Hg with concomitant retrograde bolus flow into the proximal esophagus as diagnostic for the syndrome.^50^

## 7 TECHNICAL ASPECTS OF STUDY PERFORMANCE AND INTERPRETATION

Prior to considering ambulatory manometry for everyday clinical use, one must understand the technical aspects of drifts over 24 hours and consequently make reliable measurements of esophageal pressure impossible. Therefore, we recommend against fiber optical systems such as Manoscan^™^ system for prolonged (24‐hour) pressure recording.^51^ Additionally, an ambulatory system should be lightweight and small so it can easily be carried by the patient for 24 hours. This feature requires an entirely new device compared with stationary systems. Lastly, the interpreter of ambulatory manometry recording may be faced with a learning curve. With continuous pressure monitoring, a patient can display approximately 1000‐1400 contractions over a 24‐hour monitoring period. A large number of these may represent artifacts such as coughs, straining, or burping; therefore, an accurate reader requires the skills to recognize these artifacts and adapt in their interpretation.^9^ Whereas experts have suggested exposure to a minimum of 50 esophageal high‐resolution manometry studies during training to achieve expertise in motility,^52^ the optimum threshold required for accurately interpreting 24‐hour ambulatory manometry has not yet been established.

## 8 LIMITATIONS

The application of ambulatory manometry in clinical practice is not without limitations. Firstly, authors used numerous variations in the ambulatory manometry in past studies, including differences in catheter size and transducer location. Furthermore, although normative values have been suggested for ambulatory manometry interpretation, prior studies had not universally taken these into account. These lacks in homogeneity introduce challenges when comparing and interpreting data outcomes. Secondly, available studies on ambulatory manometry are currently outdated, and therefore introduce a challenge in data interpretation. This problem likely stems from the scarcity of ambulatory manometry applied in clinical use.

## 9 CONCLUSION

In conclusion, ambulatory 24‐hour esophageal manometry makes it possible to assess temporal relationships between reflux and cough, to diagnose rumination, and to identify esophageal dysmotility as the likely cause of non‐cardiac chest pain—particularly when questions remain following traditional testing. Although the application of ambulatory esophageal manometry has mainly been a tool for experts’ centers and is not well known, it does add advantage over stationary manometry by evaluating esophageal function over an extended period of time and can add critical information to ambulatory pH‐impedance testing. Additional research will be important to further understand the value of ambulatory manometry in clinical practice and establish a current diagnostic classification system.

## CONFLICT OF INTEREST

AJB received research funding from Nutricia, Norgine, and Bayer and received speaker and/or consulting fees from Laborie, EsoCap, Diversatek, Medtronic, Dr Falk Pharma, Calypso Biotech, Thelial, Robarts, Reckitt Benckiser, Regeneron, Celgene, Bayer, Norgine, AstraZeneca, Almirall, and Allergan.

## AUTHOR CONTRIBUTIONS

AK drafted and revised the manuscript; AB revised and reviewed the manuscript; AK, JC, JO, and AB have approved the final draft submitted.
